# Editorial: Reducing Post‐Endoscopy Upper Gastrointestinal Cancer—Less Is More?

**DOI:** 10.1002/ueg2.70218

**Published:** 2026-04-18

**Authors:** Yuichi Mori, Tony C. Tham

**Affiliations:** ^1^ Clinical Effectiveness Research Group Institute of Health and Society University of Oslo Oslo Norway; ^2^ Gastroenterology Section, Department of Transplantation Medicine Oslo University Hospital Oslo Norway; ^3^ Digestive Disease Center Showa Medical University Northern Yokohama Hospital Yokohama Japan; ^4^ Division of Gastroenterology Ulster Hospital Dundonald Belfast UK

The study by Kamran et al. [1] provides an important and timely analysis of variation in post‐endoscopy upper gastrointestinal cancer (PEUGIC) rates across endoscopy providers in England. PEUGIC, defined as cancers diagnosed after a prior negative endoscopy, represents a critical quality indicator, reflecting missed diagnostic opportunities and directly impacting patient outcomes. Using linked national datasets and the National Endoscopy Database (NED), the authors compared endoscopy practices between providers in the highest and lowest quartiles of PEUGIC rates. The dataset is substantial, including over 328,000 diagnostic upper GI endoscopies performed across 54 providers between 2019 and 2020. This scale allows for robust evaluation of real‐world practice variation.

The key findings are both striking and actionable. Providers with the lowest PEUGIC rates were more likely to conduct endoscopies during training sessions, use intravenous sedation more frequently, have endoscopists with higher annual procedure, and operate with lower‐intensity lists (i.e. lower volumes in a list). Conversely, higher PEUGIC rates were associated with high‐intensity endoscopy greater involvement of non‐specialist or non‐registered endoscopists, and higher overall biopsy rates. Importantly, although overall biopsy rates were lower in better‐performing providers, adherence to guideline‐recommended biopsy protocols for high‐risk conditions (e.g., ulcers, strictures) was superior. This distinction between “more biopsies” and “appropriate biopsies” is a critical nuance. Overall, the study identifies several potentially modifiable service‐ and operator‐level factors associated with improved detection and reduced missed cancers.

This study represents a significant advance in understanding why PEUGIC rates vary so widely. Previous work [2] established the existence of variation; this analysis begins to explain it. The findings strongly support the concept that endoscopy quality is not solely dependent on individual skill but is shaped by system‐level factors, training, workload, staffing, and procedural environment. One of the most compelling findings is the association between lower list intensity and better outcomes. Providers with fewer procedures per session had lower PEUGIC rates. This aligns closely with the European Society of Gastrointestinal Endoscopy (ESGE) Quality Improvement Initiative [3], which explicitly recommends adequate time allocation, at least 20 min per diagnostic upper GI endoscopy, to ensure thorough examination. Time pressure is a well‐recognised barrier to high‐quality endoscopy. Shortened inspection times reduce mucosal assessment, limit use of advanced imaging, and may compromise lesion recognition. The ESGE further recommends a minimum inspection time of ≥ 7 min for diagnostic procedures, reinforcing that careful inspection is key to quality. The current study provides real‐world evidence supporting these recommendations.

The association between intravenous sedation and lower PEUGIC rates is also noteworthy. Sedation likely improves patient comfort, reduces movement, and allows longer, more meticulous inspection, even making endoscopists feel comfortable. While sedation is often considered primarily from a patient experience perspective, this study suggests it may also be a diagnostic quality enhancer. This also aligns with ESGE principles emphasising patient‐centred care and optimal procedural conditions, including adequate preparation and comfort, to maximise diagnostic yield [4]. It raises the question of whether sedation should be more actively promoted, not universally, but strategically, as part of quality improvement.

The finding that endoscopist volume is associated with lower PEUGIC rates reinforces existing recommendations. Endoscopists performing > 100 procedures annually,and particularly those exceeding 200, had better outcomes. This supports the concept of a volume‐outcome relationship, already recognised in other areas of endoscopy. Equally important is the observation that non‐specialist endoscopists were associated with higher PEUGIC rates. This highlights the importance of structured training, accreditation, and ongoing professional development. The ESGE initiative emphasises standardised training, reporting, and adherence to protocols as fundamental components of quality care. Interestingly, training sessions themselves were associated with better outcomes. This may reflect slower, more deliberate procedures, and a culture of reflection and learning. It challenges the assumption that training lists compromise efficiency without benefit.

The ability to recognise subtle, early neoplastic lesions in the upper gastrointestinal tract is a core competency for any practising endoscopist. Missing such lesions has clear and important clinical consequences, and detection relies as much on trained pattern recognition as it does on technology. The European Society of Gastrointestinal Endoscopy (ESGE) has set out position statements defining minimum standards and training requirements in this area [5]. These provide a useful framework, and endoscopists should be encouraged to use them to guide the development and maintenance of their diagnostic skills.

Perhaps the most nuanced finding relates to biopsy practice. At first glance, lower biopsy rates in better‐performing providers seem counterintuitive. However, the study demonstrates that targeted biopsy, specifically in high‐risk lesions, is more important than indiscriminate sampling. This directly supports ESGE recommendations such as the MAPS protocol for gastric precancerous conditions and the Seattle protocol for Barrett's esophagus [3], which emphasise structured, indication‐driven biopsy strategies.

Taken together, the findings of this study align remarkably well with the proposed quality improvement initiative [3] and training to improve detection of early neoplastic lesions. The current study also adds an important dimension: it demonstrates that variation in these domains is not theoretical, it translates into measurable differences in cancer outcomes. One of the most important implications of this article is that it underscores the need for system‐level interventions. Improving endoscopy quality is not just about training individuals but about designing services that enable high‐quality practice. This includes appropriate scheduling, workforce planning, and integration of training. To achieve this, it provides strong support for adopting comprehensive quality frameworks such as the ESGE QIC initiative. Standardising practice according to these evidence‐based measures could reduce unwarranted variation and improve cancer detection across health systems.

## Conflicts of Interest

The authors declare no conflicts of interest.

## Data Availability

The authors have nothing to report.
